# TRPML1 as a potential therapeutic target for triple-negative breast cancer: a review

**DOI:** 10.3389/fonc.2023.1326023

**Published:** 2023-12-13

**Authors:** Ying Pan, Qiancheng Zhao, Haitao He, Yubo Qi, Yujie Bai, Jia Zhao, Yiming Yang

**Affiliations:** ^1^ Department of Histology and Embryology, College of Basic Medical Sciences, Jilin University, Changchun, Jilin, China; ^2^ Department of Cell Biology, College of Basic Medical Sciences, Jilin University, Changchun, Jilin, China; ^3^ First Hospital of Jilin University, Changchun, Jilin, China

**Keywords:** triple-negative breast cancer, TRPML1, therapeutic target, cancer treatment, autophagy

## Abstract

Triple-negative breast cancer (TNBC) is the most refractory subtype of breast cancer, and effective treatments are urgently needed owing to its poor prognosis. Surgery, radiotherapy, and chemotherapy, alone or in combination, are the leading choices for TNBC therapy. Although promising approaches and procedures have emerged, several challenges, such as off-target effects, drug resistance, and severe side effects, remain to be addressed. Recently, transient receptor potential channel mucolipin 1 (TRPML1) has attracted the attention of researchers because its expression has been implicated in numerous diseases, including cancer. TRPML1 regulates biological events and signaling pathways, including autophagic flux, exocytosis, ionic homeostasis, and lysosomal biogenesis, all contributing to tumorigenesis and cancer progression. TRPML1 also functions as a building block for cancer cell growth, mitogenic signaling, priming tissues for metastasis, and activation of transcriptional programs, processes involved in several malignant tumors. This review provides an overview of breast cancer epidemiology and diagnostic techniques and then discusses the existing therapeutics. Additionally, we elaborate on the development of, and associated challenges to, TNBC diagnostics and treatment and the feasibility of TRPML1 as a therapeutic target for TNBC.

## 1 Introduction

**Figure 1:**
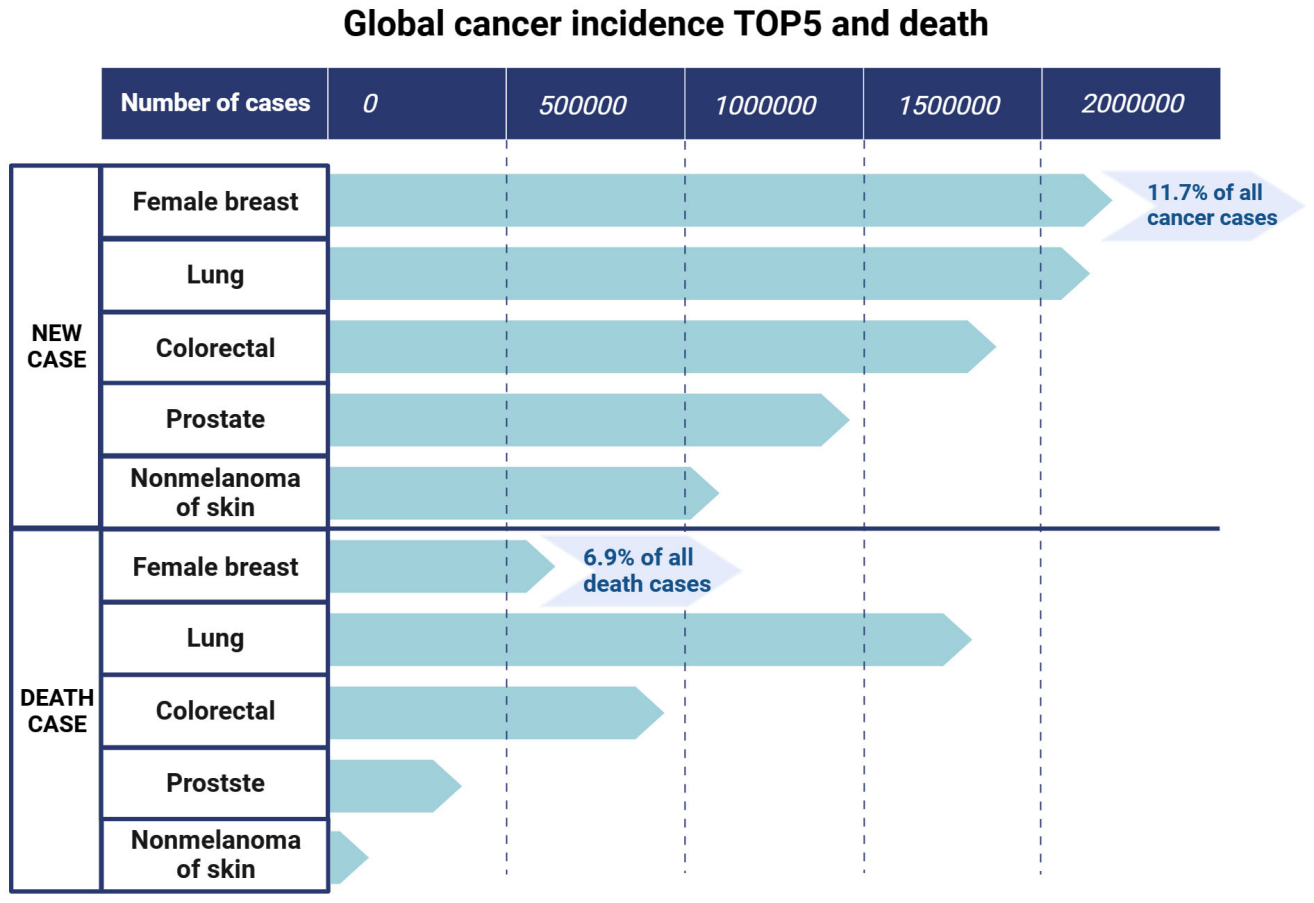
The top five new cancer cases and deaths in the world. Data (2020) are from the Global Cancer Observatory of the World Health Organization. Created with BioRender.com.

Breast cancer is a global health challenge as it is highly aggressive and commonly affects women. The latest data from 2020 show that breast cancer has surpassed lung cancer to become the most common cancer among female patients, with new cases comprising 11.7% of all cancer cases and a mortality rate of 6.9%, ranking fifth among all cancer-related deaths (1) (**Figure 1**). Although considerable progress has been made in the diagnosis and treatment of breast cancer in recent years, the issues of drug resistance, recurrence, low overall survival (OS) rate, and poor prognosis remain unresolved (2, 3).

**Figure 2:**
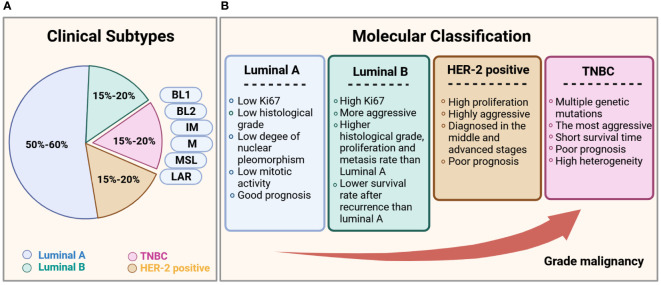
Subtypes of breast cancer. **(A)** Statistical chart of the proportion of each clinical subtype of breast cancer. **(B)** Comparison of the clinical subtypes of breast cancer, based primarily on histological features and immunohistochemical expression of ER, PR, HER2, and the proliferation marker Ki67.

Clinically, breast cancer is a heterogeneous disease with multiple subtypes (4, 5). Based on the expression of hormone receptors (HRs), namely estrogen receptor (ER), progesterone receptor (PR), and human epidermal growth factor receptor 2 (HER2). The molecular classification of breast cancer can be divided into four types: luminal A (HR^+^ [ER^+^ and/or PR^+^], HER2^-^), luminal B (HR^+^ [ER^+^ and/or PR^+^], HER2^+/-^), HER2-positive (HR^-^ [ER^-^, PR^-^], HER2^+^), and triple-negative breast cancer (TNBC; HR^-^ [ER^-^, PR^-^], HER2^-^). The molecular and biological characteristics of each subtype are presented in **Figure 2**. Among the four subtypes, TNBC is the most aggressive, with a high rate of metastasis and poor prognosis. Approximately 46% of patients with TNBC develop distant metastasis and the median survival time after metastasis is substantially shortened to only 13.3 months (6–8), with the highest death rate within five years of diagnosis (9). TNBC is also heterogeneous. Lehmann et al. (2011, 2014) divided TNBC into six subtypes, according to gene expression profiles, which include basal-like 1 and basal-like 2, immunomodulatory (IM), mesenchymal (M), mesenchymal stem-like (MSL), and luminal androgen receptor (LAR) (10, 11), and found an association between each subtype and multiple gene mutations (12). Since TNBC is the most metastatic cancer and no effective targeted therapy currently exists, early diagnosis and treatment are essential to control its development (13).

Mammography is the most important screening method for breast cancer as it facilitates early diagnosis, substantially reducing the overall patient mortality (14). In high-risk and susceptible groups, mammography is usually combined with ultrasonography or magnetic resonance imaging (MRI) for diagnosis (15), with MRI-assisted mammography being the more sensitive approach (16). For detecting distant metastases, the sensitivity and specificity of positron emission tomography (PET)/CT scans are considerably higher than those of conventional imaging examinations and have a higher diagnostic value for prognostic risk stratification (17), with 18-fluoro-deoxyglucose PET/CT explicitly used to detect axillary lymph node metastases (97%) (18).

## 2 Current therapies for breast cancer

Advances in imaging, treatment, and posttreatment care over the past decade have provided a range of treatment options for breast cancer. For non-metastatic breast cancer, surgical intervention is the mainstay treatment. Notably, no considerable difference in OS has been observed between breast-conserving surgery plus radiotherapy and total mastectomy (19, 20). The mainstay treatment for hormone-responsive (HR^+^, HER2^-^) breast cancer is endocrine therapy, which blocks the action of estrogen at the receptor level or inhibits estrogen production using selective ER modulation agents, aromatase inhibitors, luteinizing hormone-releasing agonists, and cyclin-dependent kinases 4 and 6 inhibitor for resistant patients (21, 22). HER2 inhibitors are used primarily to treat the HER2 subtype of breast cancer, along with additional small-molecule tyrosine kinase inhibitors, such as afatinib and neratinib (23). These drugs can cross the blood-brain barrier and reduce the rate of brain metastasis in patients with HER2^+^ breast cancer (24). Interestingly, combined antibody–drug conjugates (ADC) in high-risk patients achieves a pathological complete response (pCR), suggesting that a proportion of HER2-positive tumors can be eradicated without chemotherapy (25).

As TNBC is not sensitive to estrogen-sensitive endocrine therapy or HER2-targeting drugs, the chemotherapy-based systemic therapy is the primary systemic treatment option; however, its efficacy is limited, and the associated prognosis is poor (26). Neoadjuvant systemic therapy (NAST) is becoming the standard treatment for TNBC in the early stages of the disease (12, 19, 27). However, no standard chemotherapeutic regimen for relapsed/refractory patients with TNBC is currently available. Response rates of TNBC increase with chemotherapy drug combinations compared to the use of single agents; however, this increase was at the expense of increased cytotoxicity (28, 29). Currently, prolonging the survival of patients with TNBC is not universally or effectively possible.

## 3 Molecularly targeted therapies for TNBC

Considering chemotherapy’s nonspecificity and high toxicity, developing targeted therapeutic approaches and regimen combinations is crucial for improving TNBC prognosis. With the continued elucidation and characterization of the biological nature of TNBC, molecularly targeted strategies have been identified, including DNA repair pathway inhibitors, immunotherapy, anti-angiogenic agents, ADCs, and phosphatidylinositol 3-kinase (PI3K)/protein kinase B (AKT)/mammalian target of rapamycin (mTOR) inhibitors. We listed clinical medicines and their advantages and disadvantages in **Table 1**.

**Table 1:** Mechanism and characteristics of molecular targeted therapy for triple-negative breast cancer.

Therapeutic method	Mechanism	Drug	Advantages	Disadvantages
Platinum Agents	Targets DNA repair pathway, cross-links DNA damage-mediated apoptosis of tumor cells	Carboplatin Cisplatin	High pCR	Dose-limiting toxicity (30, 31)
PARPis	Targets DNA repair pathway, anti-proliferative and proapoptotic properties	Olaparib Talazoparib Aparib Veliparib Rucaparib Niraparib	Chemosensitizers Radiosensitizers	Drug resistance (32–37)
PD-1/PDL-1 CPI	Prevent tumor cells from evading the immune system by blocking PD-1/PD-L1-mediated immune cell suppression	Nivolumab Avelumab Pembrolizumab Atezolizumab Durvalumab	Combined with PARPi exhibit synergistic activity	Neutropenia Febrile neutropenia hypertension immune-related hypothyroidism (38–42)
Anti-angiogenic agents	Disrupts tumor angiogenesis, inhibiting tumor growth and migration	Bevacizumab	Increase pCR and enhance PD-1 antibody efficacy	Hypertension Neutropenia Mucositis Postoperative complications (43–45)
ADCs	Target specific antigens on the surface of tumor cells; internalized through receptor-mediated endocytosis and cleaved to release a payload drug that induces immunogenic cell death through direct cytotoxicity	Sacituzumab govitecan	Efficacy in BRCA1 and BRCA2 mutant carriers	1. Cannot be administrated alone due to unacceptable toxicity 2. Neutropenia, diarrhea, nausea, anemia (46–48)
		Ladiratuzumab vedotin	Safety and efficacy	Fatigue nausea alopecia peripheral neuropathy (49)
PI3k/AKT/mTOR Inhibitors	Participates in the regulation of angiogenesis, tumor proliferation, and inhibition of apoptosis	Everolimus Buparlisib Alpelisib Ipatasertib Capivasertib	Combination of everolimus with chemotherapy is well-tolerated	Combination of everolimus with chemotherapy does not improve disease-free or overall survival or pCR (5, 50–54)

pCR, pathological complete response; PARPis, poly ADP-ribose polymerase inhibitors; CPI, checkpoint inhibitors; OS, overall survival; ADCs, antibody-drug conjuga.

Generally, TNBC associated with BRCA1 or BRCA2 mutant is currently the only type sensitive to targeted therapy, approximately 5% of patients with breast cancer carry germline mutations in BRCA1 or BRCA2 (55, 56). However, the efficacy and safety of DNA repair pathway inhibitors differ concerning their potency and cytotoxicity regarding their ability to prevent poly ADP-ribose polymerase from directing DNA repair (33).

Compared to other types of breast cancer, TNBC has a higher mutation load, stronger immunogenicity, and more immune cell infiltration, implying that immunotherapy may be a viable treatment option for TNBC (57). Indeed, adding the programmed cell death protein 1 (PD-1)/programmed death-ligand (PD-L1) pathway blockers prevent tumor cells from evading the immune system by blocking PD-1/PD-L1-mediated immune cell suppression (38). Adding a PD1/PD-L1 blockade to NACT considerably increases the pCR rate in patients with TNBC with a good safety response, especially in patients with a high risk of recurrence (39). Therefore, immunotherapy may be an ideal direction for future TNBC treatment, particularly in combination with chemotherapy.

ADCs target specific antigens on the surface of tumor cells. They are internalized and cleaved to release a payload drug that drives antitumor activity and induces immunogenic cell death through direct cytotoxicity. The main ADCs currently used in TNBC are sacituzumab govitecan and ladiratuzumab vedotin. Other ADC drugs are also under development, including an anti-intracellular adhesion molecule 1 ADC; however, drug resistance continues to occur due to antigen-associated drug resistance, internalization failure, and impaired lysosomal function (30, 58).

Mutations in components of the PI3K/AKT/mTOR signaling pathway can result in its persistent activation, which is often observed in different malignancies (59, 60). The mutation frequency of PIK3CA (a PI3K subunit) in TNBC is approximately 10%, and activation of related pathways is more common in LAR and M subtypes (61). In clinical trials, drugs such as the pan-PI3K inhibitors, AKT inhibitors, and mTOR inhibitors have achieved satisfactory results (5, 50). However, in patients with early TNBC, adding the PI3K/AKT pathway inhibitor ipatasertib to paclitaxel-based neoadjuvant regimens does not improve pCR, suggesting that more precise guidance is needed for the use of this type of drug (51).

Although these targeted therapies prolong the development time of TNBC to varying degrees or temporarily prolong the survival period, there is still no effective solution to prevent disease recurrence and drug resistance. Currently, methods that can effectively prolong the survival of patients with TNBC, improve prognosis, and control its progression with mild adverse reactions are lacking. Furthering our understanding of the microscopic nature of TNBC, identifying novel therapeutic targets, and precisely defining a patient population suitable for existing targeted drug therapies are necessary to develop effective TNBC treatments.

## 4 TRPML1 as a potential therapeutic target for TNBC

**Figure 3:**
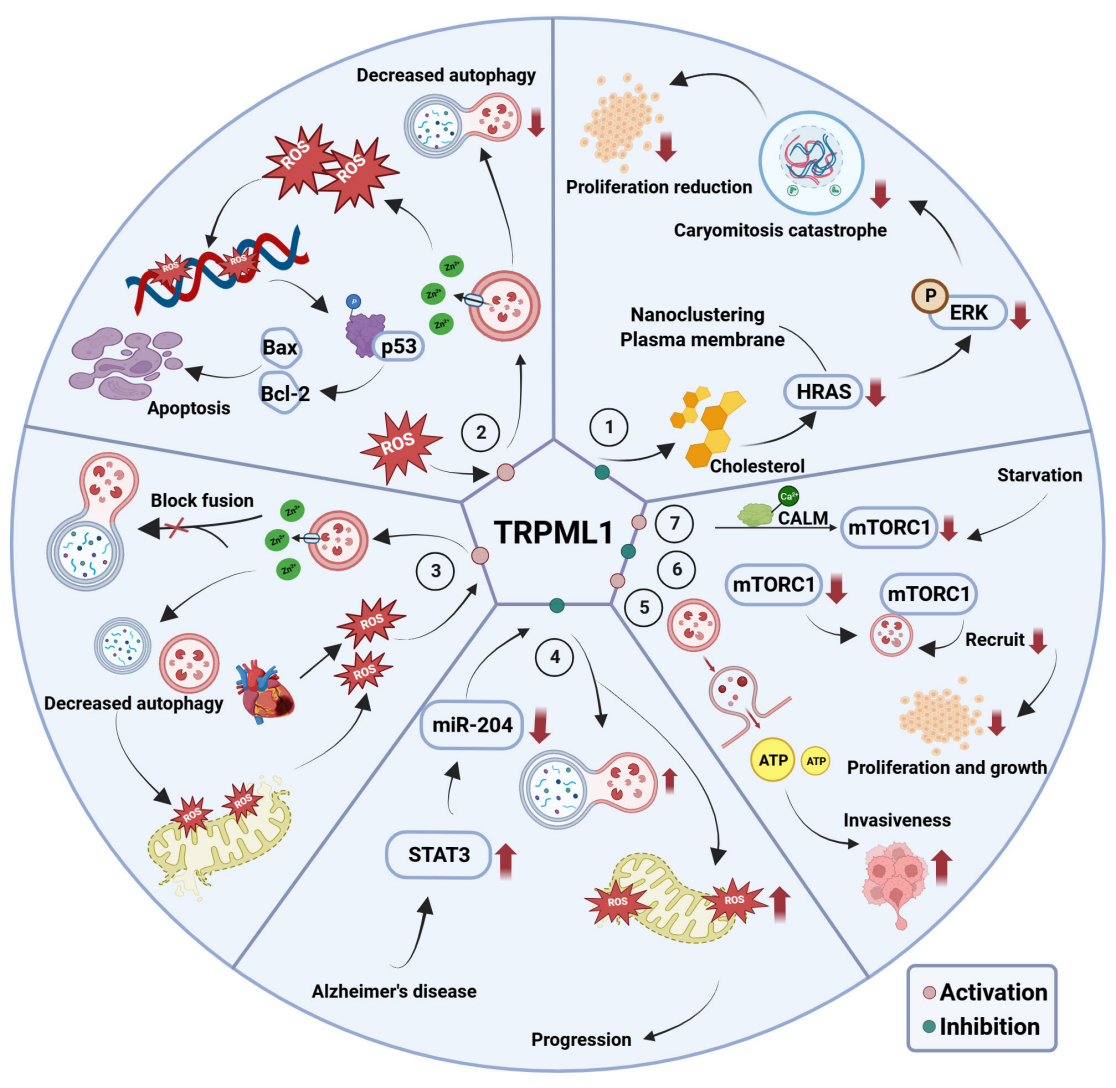
The mechanism of TRPML1’s involvement in associated diseases. ① TRPML1 inhibition disrupts cholesterol distribution and abundance, attenuating HRAS nanoclustering and plasma membrane abundance. This evokes unrestricted ERK phosphorylation, inhibitory mitogenic pathways and decreased cell proliferation; ② In melanoma and glioblastoma cells, activation of TRPML1 releases lysosomal Zn^2+^ to disrupt the fusion between autophagosomes and lysosomes, thereby triggering ‘autophagy inhibition, disrupted mitochondria turnover, ROS elevation, DNA damage, p53 activity, apoptosis’ axis, and ultimately triggering mitochondrial-mediated apoptosis; ③ TRPML1 is activated secondary to ROS elevation following ischemia/reperfusion, which in turn induces the release of lysosomal Zn^2+^ into the cytosol. This disrupts the fusion between autophagosomes and lysosomes, as well as mitochondria turnover, and further excessive ROS release; ④ In AD, high expression of miR-204 targets TRPML1, thus promoting ROS production and inhibiting mitochondrial autophagy, thereby promoting AD progression; ⑤ Elevated TRPML1 in MDA-MB-231 cell promotes cell invasion through enhanced lysosomal ATP release via lysosomal exocytosis; ⑥ Inhibition of TRPML1 reduction in mTORC1 activity as well as attenuation of lysosomal mTORC1 recruitment and decreased cell proliferation; ⑦ Activity of mTORC1 decreases during nutrients starvation, and then causing TRPML1 to be activated to release Ca^2+^ which binds to CALM, leading to mTORC1 recruitment onto lysosomes.

A potential target for treating cancer has emerged in recent years—transient receptor potential channel mucolipin 1 (TRPML1). As a lysosomal channel protein, TRPML1 can permeate various cations, including calcium ions, maintain cell homeostasis, regulate lysosomal adaptation, exocytosis, autophagy, and other functions, and mediate cancer cell growth (62–64). The mechanisms through which TRPML1 contributes to disease pathogenesis are summarized in **Figure 3**. In particular, TRPML1 plays a critical role in TNBC (65). Xu et al. reported that TRPML1 is specifically up-regulated in TNBC and controls cancer cell survival by promoting mTORC1 activity. Meanwhile, decreased cell proliferation was observed in TRPML1-knockdown (KD) MDA-MB-231 cells. Moreover, TRPML1-KD MDA-MB-231 cells exhibit attenuated extracellular ATP content and invasive capacity, indicating that TRPML1 facilitates TNBC metastasis by enhancing extracellular ATP release. Similar results were reported by Shekoufeh et al. (66), who showed that TRPML1 promotes breast cancer cell line survival by supporting mitochondrial function and cellular metabolism. Specifically, TRPML1 KD inhibits TNBC mitochondrial respiration, glycolysis, and ATP production, leading to reduced proliferation, promotion of cell cycle arrest, and apoptosis with enhanced global and mitochondrial ROS in MDA-MB231 and HS578PT cells. Furthermore, TRPML1 downregulation enhances the sensitivity of TNBC cells to chemotherapy drugs. These studies strongly suggest that TRPML1 is a therapeutic target in TNBC. Additionally, TRPML1 is reportedly involved in various tumor-associated processes.

### 4.1 TRPML1 is a biomarker of various tumors

Tumor cells driven by HRas proto-oncogene GTPas (HRAS), a small GTPase member of the Ras superfamily, are more sensitive to TRPML1 inhibition, resulting in reduced growth, invasion, and proliferation of cancer cells (67). High TRPML1 expression is markedly associated with highly malignant cancers (68). The expression of TRPML1 in metastatic melanoma is higher than that in benign melanoma and normal skin tissue (69). Patients with pancreatic ductal adenocarcinoma with high expression of TRPML1 also have a poorer postoperative prognosis (70). Additionally, TRPML1 plays a role in breast cancer, especially in TNBC with high metastasis, high recurrence, and poor prognosis, as high TRPML1 expression is closely associated with the occurrence, development, and prognosis of TNBC (65, 66). Therefore, TRPML1 is an notable candidate for research on targeted therapy of TNBC.

### 4.2 TRPML1 regulates mTOR

mTOR regulates cell growth, proliferation, and metabolism in response to multiple substances, including growth factors, hormones, and energy (ATP). mTOR is a major signaling pathway for cancer cell growth and survival (71). Patients with phospho-mTOR expression showed substantially worse OS and recurrence-free survival rates. The association between mTOR activity and TNBC has attracted the attention of many researchers because mTOR overactivation is common in many cancers, including breast cancer, especially TNBC, as more activated mTOR is observed in TNBC than in other breast cancer subtypes (72).

As mentioned earlier, the mTOR inhibitor everolimus has entered the clinical trial stage for efficacy evaluation. However, since mTOR is widely involved in various biological processes in most cells, mTOR inhibitors do not provide substantial benefits to patients with cancer. Lysosomal calcium signaling plays a vital role in regulating the mTOR-dependent autophagy pathway, and TRPML1 provides negative feedback regulation of mTOR through calmodulin (73). Therefore, based on the regulatory effect of TRPML1 on mTOR, the development of regulatory drugs targeting TRPML1 activity to achieve tumor suppression may provide greater specificity and better therapeutic effects than mTOR inhibitors.

### 4.3 TRPML1 regulates autophagy

TRPML1 is critical for autophagy regulation. For example, upregulation of TRPML1 in Alzheimer’s disease (AD) inhibits reactive oxygen species (ROS) generation and mitophagy in AD through the signal transducer and activator of transcription 3 (STAT3) pathway, which is implicated in many aspects of autophagy (74). Pharmacological or genetic inhibition of TRPML1 restores impaired cardiomyocyte autophagy following myocardial ischemia/reperfusion injury (75). Concerning tumor cell survival in a more complex environment, TRPML1 activation inhibits autophagy in various cancer cell lines by disrupting the fusion between autophagosomes and lysosomes. More importantly, TRPML1-mediated inhibition of autophagy inhibits cancer cell growth by triggering apoptosis (62). The investigation aimed at elucidating how autophagy regulates apoptosis has revealed that TRPML1-induced autophagy inhibition triggers mitochondrial turnover disruption, leading to ROS elevation, which causes severe DNA damage in cancer cells (76). As mentioned previously, platinum drugs and PARPi are effective in treating TNBC by inhibiting DNA repair, however, drug resistance and side effects exist. Thus, TRPML1 may represent a new target for TNBC intervention via DNA repair pathway strategies.

In contrast, TRPML1 expression also has a protective survival effect in patients with glioblastoma (77); loss of TRPML1 impairs melanoma growth, reduces cell proliferation, and attenuates cell viability (78). Therefore, the conflicting roles of TRPML1 in regulating autophagy suggest that its role in tumors deserves further exploration. Regardless, TRPML1 plays an undeniably important role in mediating the occurrence and development of tumors through autophagy. Since TRPML1 is highly expressed in TNBC and is tightly linked to mTOR-dependent autophagy, we speculate that TRPML1 may affect TNBC by regulating autophagy.

### 4.4 Application of TRPML1 agonists in various treatments

Many studies have targeted TRPML1 to seek novel therapeutic methods for various diseases. In Parkinson’s disease, *Artemisia annua* leaf extract rescues neurons and elicits a neuroprotective effect by stimulating TRPML1 activity, promoting autophagy/mitochondrial autophagy, and upregulating survival pathways (79). In AD, endosomal–autophagic–lysosomal system defects in AD neurons are rescued by the reactivation of TRPML1 using the TRPML agonist ML-SA1 (80). Furthermore, treatment with a specific activator of TRPML1, ML1-SA1, decreases mitochondrial membrane potential through excessive activation of TRPML1 in liver cancer cells, resulting in mitochondrial damage and dysfunction (81). The same phenomenon was observed when TRPML1 was knocked out using the CRISPR/Cas9 gene editing technique. Studies on liver cancer have shown that TRPML1-mediated exocytosis promotes metastasis (81, 82). In malignant melanoma, TRPML1-induced inhibition of autophagy inhibits cancer metastasis by stimulating the ROS-mediated TP53/p53 pathway (83). TRPML1 agonist, ML-SA5, induces substantial cell death in malignant melanoma cell lines but fully preserves normal melanocytes *in vitro*. However, it fully preserves normal melanocytes (69). Another *in vivo* study showed that ML-SA5 improves muscle atrophy in Duchenne muscular dystrophy mice *in vivo* by promoting myosin repair (84). These studies demonstrate that clinical drugs targeting TRPML1 are possible. Further experiments should be conducted to identify drugs or techniques that change the function or expression of TRPML1 to clarify the specific mechanism of TRPML1 regulation in TNBC and other tumors and diseases for further development of novel treatments.

## 5 Discussion

TNBC accounts for only 15–20% of all breast cancer cases. However, it remains a challenging disease due to its poor cellular differentiation, high heterogeneity, and rapid metastasis (85). Clinically, in the early stages of TNBC, complete surgical excision with NAST is the standard treatment. Anthracycline/taxane-based combination chemotherapy is the first-line treatment for patients with metastatic or advanced TNBC. Although developing precise targets and inhibitors for TNBC has gradually attracted attention, their clinical effectiveness and safety require further verification. Finding new therapeutic targets and treatment methods remains an essential topic in TNBC research.

TRPML1 is an important biomarker of various tumors, and treatment with small-molecule activators that target TRPMLI has therapeutic potential in neurodegenerative diseases and various tumors. High expression of TRPML1 is closely associated with the occurrence, development, and prognosis of TNBC, and TRPML1 regulates multiple biological events and molecular targets associated with TNBC pathology. Therefore, TRPML1 could be a potential target for TNBC-targeted therapy. With the deepening of our understanding of the molecular characteristics and tumor microenvironment structure of TNBC and the development of new detection methods, more accurate and effective targeted treatment methods will emerge, resulting in improved curative effects in patients with TNBC.

## Author contributions

YP: Writing – original draft. QZ: Writing – original draft. HH: Funding acquisition, Writing – review & editing. YQ: Writing – review & editing. YB: Writing – review & editing. JZ: Conceptualization, Writing – review & editing. YY: Conceptualization, Funding acquisition, Supervision, Writing – review & editing.
